# Mitochondrial Quality Control in COPD and IPF

**DOI:** 10.3390/cells7080086

**Published:** 2018-07-24

**Authors:** Hiromichi Hara, Kazuyoshi Kuwano, Jun Araya

**Affiliations:** Division of Respiratory Diseases, Department of Internal Medicine, The Jikei University School of Medicine, Tokyo 105-8461, Japan; kkuwano@jikei.ac.jp (K.K.); araya@jikei.ac.jp (J.A.)

**Keywords:** mitochondria, mitochondria dynamics, mitophagy, chronic obstructive pulmonary disease (COPD), idiopathic pulmonary fibrosis (IPF)

## Abstract

Mitochondria play important roles in the maintenance of intracellular homeostasis; hence, the quality control of mitochondria is crucial for cell fate determination. Mitochondria dynamics and mitochondria-specific autophagy, known as mitophagy, are two main quality control systems in cells. Mitochondria fuse to increase energy production in response to stress, and damaged mitochondria are segregated by fission and degraded by mitophagy. Once these systems are disrupted, dysfunctional mitochondria with decreased adenosine triphosphate (ATP) production and increased reactive oxygen species (ROS) production accumulate, affecting cell fate. Recently, increasing evidence suggests that the dysregulation of mitochondria quality control is pathogenic in several age-related diseases. In this review, we outlined the role of mitochondria quality control systems in the pathogenesis of age-associated lung diseases, chronic obstructive pulmonary disease (COPD) and idiopathic pulmonary fibrosis (IPF).

## 1. Introduction

**Figure 1:**
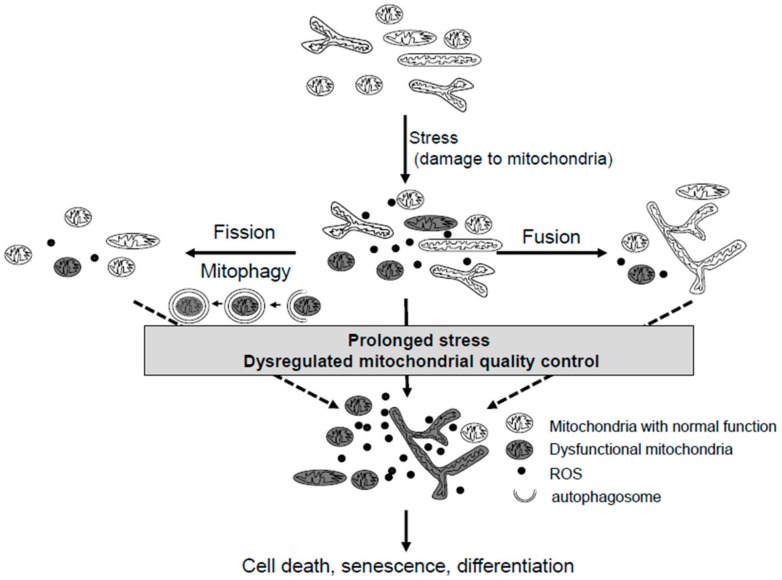
The mitochondrial quality control systems in cells. Under mild stress, mitochondrial fusion dilutes the damage of dysfunctional mitochondria, or fission leads to the segregation and removal of damaged mitochondria by mitophagy. However, if the stresses are severe and prolonged or if the mitochondrial quality control systems are dysregulated, these adaptive responses are overwhelmed by the stress, affecting the cell fate.

Mitochondria play multiple roles in cells [1,2]. Mitochondria are involved in not only energy production by oxidative phosphorylation but also in a variety of vital metabolic processes to maintain intracellular homeostasis [1,2]. Indeed, mitochondria play important roles in the heme biosynthesis, regulation of intracellular calcium, and fatty acid synthesis. Hence, mitochondrial dysfunction leads to cell dysfunction, including cell death [3,4]. To prevent the accumulation of mitochondrial damage, cells have several intracellular machineries [5,6]. Mitochondrial dynamics [3] and mitochondria-specific autophagy, known as mitophagy [7], are two main machineries that reduce mitochondrial damage and maintain intracellular homeostasis. Mitochondria are continuously exposed to environmental and intracellular stresses including environmental toxins like cigarette smoke, reactive oxygen species (ROS) that cause mitochondrial DNA damage, amino acid depletion, and unfolded proteins. To overcome these stresses, mitochondrial dynamics and mitophagy work together. However, when the stresses overwhelm these quality control systems, dysfunctional mitochondria with decreased adenosine triphosphate (ATP) production and increased ROS production accumulate. Accumulation of dysfunctional mitochondria subsequently disrupts intracellular homeostasis and changes the cell fate (Figure 1), which contributes to the pathogenesis of several diseases [8].

Mitochondrial functions decrease with age, while mitochondrial DNA mutations increase with age [9,10,11]. Some mitochondrial DNA mutations cause mitochondrial dysfunction, which is crucial for progeroid manifestations in mice through stem cell dysfunction with excessive ROS [12]. These observations suggest that mitochondrial dysfunction plays critical roles in the development of the aging phenotype. Hence, mitochondrial quality control systems might also be important in the development of the aging phenotype and age-associated diseases through regulating mitochondrial function. Indeed, increasing evidence suggests that the disruption of mitochondrial quality control and subsequent mitochondrial dysfunction is closely associated with some age-associated diseases. Pathogenic roles of dysregulated mitochondrial quality control systems in neurodegenerative disorders have been investigated intensively, and the underlying mechanisms have been elucidated to a great extent [8]. Recently, several lines of evidence suggest that the dysregulation of mitochondrial quality control systems also contributes to the pathogenesis of age-associated lung diseases, chronic obstructive pulmonary disease (COPD) [13,14,15,16,17] and idiopathic pulmonary fibrosis (IPF) [17,18,19]. In this review, we outlined the role of the mitochondria quality control system on COPD and IPF.

## 2. Mitochondrial Quality Control Systems

### 2.1. Mitochondria Dynamics

Mitochondria are dynamic organelles which continuously change their shapes by fusion and fission [4]. Mitochondrial dynamics are regulated by the balance of expression levels between fission and fusion proteins. 

Fusion is mediated by membrane-anchored proteins, mitofusin (MFN)-1,2, and optic atrophy (OPA)-1. MFNs and OPA-1 promote the fusion of outer mitochondrial membranes and inner mitochondrial membranes, respectively. These fusion proteins usually work coordinatingly. The deficiency of fusion proteins leads to the fragmentation of the mitochondria [20].

Fission is mediated by cytosolic dynamin, dynamin-related protein 1 (Drp1), Fission1 protein (Fis-1), Mitochondrial Fission Factor (MFF) and other proteins. Drp-1 plays major roles in these processes, recruited to mitochondria from cytosol. The deficiency of Drp-1 leads to hyperfusion of the mitochondria [21].

When cells are exposed to mild stresses, mitochondria become elongated by promoting fusion (stress-induced mitochondrial hyperfusion or SIMH) [4]. SIMH requires uncleaved forms of OPA-1 (L-OPA-1), MFN1, and the mitochondrial inner membrane protein SLP-2 [22]. SLP-2 maintain OPA-1 in the uncleaved form. Mitochondrial fusion induced by mild stresses increases metabolic efficiency by enhancing ATP production and minimizing cellular damage by allowing functional mitochondria to complement dysfunctional mitochondria by diffusion and sharing of components between organelles. Hence, mitochondrial hyperfusion is an adaptive response in physiological conditions and is related to increased survival and apoptosis resistance. In contrast, under severe stress conditions, badly damaged mitochondria will contaminate other mitochondria and poison a healthy mitochondrial network if they are allowed to rejoin the network. Hence, several mechanisms are at work to stop this from happening [23]. Dysfunction of mitochondria induces OPA-1 processing through metalloprotease and the degradation of MFN through the ubiquitin-proteasome system, which leads to mitochondrial fragmentation. Fragmented mitochondria are prone to being eliminated [24].

Under severe or prolonged stress, mitochondrial morphology does not necessarily reflect mitochondrial functions. Mitochondrial functions depend on the stress type, stress severity, and the levels of other mitochondrial quality control systems (i.e., mitophagy) rather than simple morphology. Indeed, while adaptive elongated mitochondria demonstrate increased mitochondrial function, cigarette smoke-induced elongated mitochondria (with decreased mitophagy) demonstrated low mitochondrial functions and increased ROS production [25]. Whether mitochondria are elongated or fragmented, ROS from damaged mitochondria determines the cell fate [15,25,26], suggesting that mitochondrial function is more important for cell fate determination than morphology.

### 2.2. Mitophagy

Autophagy is a process of lysosomal self-degradation that helps maintain a homeostatic balance between the synthesis, degradation, and recycling of cellular proteins and organelles. It is classified into non-selective autophagy and selective autophagy [27,28]. Selective autophagic degradation of damaged mitochondria is termed mitophagy [29]. Mitophagy plays an important role in the quality control of mitochondria and is highly associated with cell fate, including senescence [13], apoptosis [30], and necroptosis [14].

**Figure 2:**
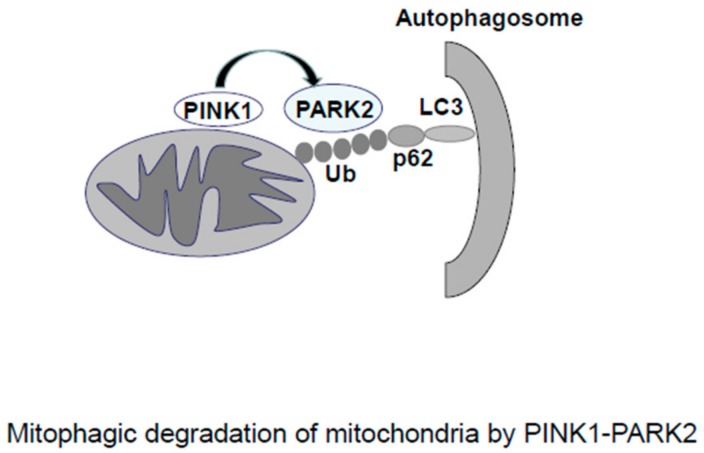
The regulation of mitophagy by the PTEN-induced putative kinase 1 (PINK1)–PARK2 system. When mitochondria are damaged by severe stresses, stress-induced membrane depolarization stabilizes PINK1, and PINK1 recruits PARK2. PARK2 ubiquitinates mitochondrial proteins, and the ubiquitinated proteins connect to microtubule-associated protein 1 light chain 3 (MAP1LC3/LC3) on phagophores through the adaptor protein SQSTM1/p62, resulting in autophagosome formation.

The main processes of mitophagic degradation of mitochondria are as follows (Figure 2) [29]. PTEN-induced putative kinase 1 (PINK1) is a serine/threonine kinase that is constantly cleaved and degraded in healthy mitochondria. When mitochondria are damaged by severe stresses, stress-induced membrane depolarization stabilizes PINK1, and PINK1 recruits PARK2, an E3-ubiquitin ligase, to the mitochondria. PARK2 ubiquitinates mitochondrial proteins, including MFNs, and ubiquitinated proteins connect to microtubule-associated protein 1 light chain 3 (MAP1LC3/LC3) on phagophores through adaptor protein SQSTM1/p62.This results in autophagosome formation. MFN degradation promotes fission, leading to fragmentation of mitochondria and the subsequent autophagic degradation. 

Mitophagy also occurs independently of PARK2. Smurf1 and Mul1, which are other E3 ubiquitin ligases, can ubiquitinate mitochondrial proteins which interact with SQSTM1/p62 and promote autophagosome formation and mitophagy [5]. Protein ubiquitination is not indispensable for mitophagy. Instead of adaptor protein p62, BNIP3, NIX, FUDC1, or cardiolipin in mitochondria directly bind to LC3 and form autophagosomes on damaged mitochondria [5]. However, the roles of PARK2-independent mitophagy in cells remain to be understood.

Mitophagy is associated with mitochondrial morphology [4]. Mitophagy induction is associated with separating fragmented mitochondria. Large fused mitochondria are spared from mitophagic degradation. Indeed, mitochondrial elongation during starvation protects mitochondria from autophagosomal degradation, which could permit mitochondria to maximize energy production although autophagy in the whole cell is upregulated [31].

### 2.3. Mitochondrial Biogenesis

Coordination between mitochondrial biogenesis and mitophagy is essential for proper mitochondrial homeostasis, and the disequilibrium between mitochondrial biogenesis and mitophagy causes the deterioration of cellular function and cell death [32].

Cells can increase energy production by increasing mitochondrial biogenesis in response to internal and external stresses. Mitochondrial biogenesis is a complex cellular process, which is regulated by the master regulator, peroxisome proliferator-activated receptor gamma coactivator 1-alpha (PGC-1α). In general, mitochondrial biogenesis is an adaptive response to increased energy demand. However, increased mitochondrial biogenesis may result in two opposite outcomes for cells depending on the mitochondrial function [33]. Increased energy production with increased mitochondrial biogenesis is beneficial for intracellular homeostasis and cell survival [34]. However, increased mitochondrial biogenesis with decreased function or with excessive ROS production disrupts intracellular homeostasis, inducing cell death or cellular senescence [35]. Increased “healthy mitochondria” (normal Oxygen Consumption Rate (OCR) and membrane potential) are beneficial for cells. However, an increased number of dysfunctional mitochondria are harmful to cells. 

### 2.4. Other Mitochondrial Quality Control Pathways

Mitochondria-derived vesicles (MDVs) and mitochondria spheroid formation are other pathways for mitochondrial quality control [5]. 

Mitochondria bud vesicles (a kind of MDV) that include oxidized mitochondrial protein and lipids are finally degraded by the lysosome. MDV formation was induced by oxidative stress independent of the loss of membrane potentials. MDVs regulate mitochondrial quality faster than mitophagy. Hence, the MDV formation may be of significance under milder mitochondrial stress conditions.

The mitochondria spheroid formation is induced by severe stress in the absence of PARK2. ROS prevent the mitofusin-mediated refusion of fragmented mitochondria, directing them to a mitochondrial spheroid formation [36]. They are positive for lysosomal proteins, possibly through the fusion with a lysosome, suggesting that this system also contributes to mitochondria quality control.

However, further studies are required to elucidate the role, interaction, and regulation of these pathways.

## 3. COPD

### 3.1. Mitochondrial Quality Control in COPD

Chronic obstructive lung disease (COPD) is a chronic lung disease characterized by airway obstruction and emphysema. Cigarette smoke is one of the main causes of this disease, and it directly affects epithelial cells. Cellular senescence and cell death, including apoptosis, are increased in COPD, which play important roles in COPD pathogenesis [37,38]. ROS, especially ROS derived from damaged mitochondria, are major determinants for cigarette smoke-induced cellular senescence in vitro [15]. Hence, it is plausible that mitochondrial quality control systems play critical roles in COPD pathogenesis [39].

### 3.2. Mitochondrial Dynamics in COPD

Diverse effects of cigarette smoke on mitochondrial morphology are observed in vitro. Nontoxic doses of cigarette smoke extract (CSE) induce mitochondrial elongation accompanied by increased metabolic activity without mitochondrial damage and ROS production in mouse alveolar cells [40]. Mitochondrial elongation, mediated by the increased expression of MFN, is regarded as an adaptive pro-survival response. Low dose CSE also induces mitochondrial elongation in different cell types, including fibroblast and small airway epithelial cells [25].

In contrast, exposure to more toxic doses of CSE induces mitochondrial fragmentation in human bronchial epithelial cells (HBEC) [13,14,15]. This morphological change is mediated by the recruitment of Drp-1 to the mitochondria [15]. Interestingly, in this experimental condition, CSE induces mitochondrial elongation at 24 h after exposure, but fragmentation is predominant at 48 h (unpublished data). Under prolonged CSE exposure, mitochondria change their shape from a fused form to a fragmented form. If fragmented mitochondria with a decreased function and increased ROS production were not eliminated, they induce cellular senescence [15]. Higher doses of CSE induce cell death [41].

Long-term exposure to CSE causes more complex changes in mitochondrial morphology. A human bronchial epithelial cell line exposed to 6 months of long-term CSE demonstrated morphologically different types of mitochondria, including elongation and fragmentation [42], which might lead to different cellular phenotypes appearing after long-term CSE exposure [43]

Similar to different types of mitochondria detected in cells exposed to long-term CSE, both elongated and fragmented mitochondria are increased in human COPD lung tissue [15,42]. This phenomenon might reflect the co-existence of mitochondria exposed to different levels of chronic cigarette smoke in COPD or diverse mitochondrial and cellular responses to cigarettes during long-term exposure in COPD. 

Prohibitin1 is an inner mitochondrial membrane protein that forms protein complexes, which are essential components of the mitochondrial fusion machinery. Decreased levels of prohibitin1 might affect mitochondrial morphology in COPD [44] 

### 3.3. Mitophagy in COPD

CSE could induce mitophagy with an increased fragmentation of mitochondria [13] or inhibit [25] mitophagy with elongated mitochondria in a cell-type and CSE dose-dependent manner in vitro. The imbalance of mitochondrial morphology toward fragmentation in the former study indicates that the given stresses are severe and mitophagy is not sufficient to eliminate damaged fragmented mitochondria prone to degradation by mitophagy. Indeed, the accumulation of CSE-induced fragmented mitochondria is associated with decreased function and increased ROS production [13,15], despite increased mitophagy. Whether mitophagy is induced or inhibited, the role of mitophagy in CSE-induced cellular senescence is similar in these studies. The enhancement of mitophagy by PARK2 overexpression inhibited the CSE-induced cellular senescence, while the inhibition of mitophagy by PARK2 inhibition accelerated the CSE-induced cellular senescence. Mitophagy protects cells from CSE-induced cellular senescence, and insufficient mitophagy causes cellular senescence. ROS from damaged mitochondria play critical roles in CSE-induced cellular senescence. In accordance with the results of in vitro data, PARK2 knockout enhanced airway wall thickening with emphysematous changes following CS exposure in COPD mouse models (Araya J et al., manuscript in submission). 

In COPD lung tissue, PARK2 expression was decreased [13] and PINK1 expression was increased with the accumulation of damaged mitochondria [42]. These observations suggest that impaired mitophagy contributes to COPD pathogenesis. 

In contrast to these reports, Mizumura et al. demonstrated that the acceleration of mitophagy leads to cell death in chronic CS-exposed mice. PINK1 was increased in chronic CS-exposed mice, and PINK1 knockout mice were protected against airspace enlargement during CS exposure [14]. They finally concluded that the increased expression of PINK1 in COPD lung tissue indicated mitophagic activation in COPD, and excessive mitophagy activation by a toxic dose of CSE might induce cell death, leading to emphysema. They also demonstrated that the expression of the necroptosis regulator, RIP 3, was increased in COPD patients and emphasized the importance of mitophagy dependent necrotic cell death in the pathogenesis of COPD. 

The discrepancy of this study and former studies might result from a difference in the experimental conditions. The role of stress-induced mitophagy in cellular homeostasis might depend on the type and amount of the stress. 

Mitophagy is a dynamic process, hence, an accurate evaluation of the mitophagy status in tissues is difficult. For example, PINK1 accumulation is regarded as a result of suppressed mitophagy in several studies [25], but the same phenomenon was regarded as mitophagy induction in another study [14]. Further studies are needed to elucidate the mitophagic status in a COPD lung. However, recently, we reported that PARK2 (not PINK1) is the dominant regulator of CSE-induced mitophagy in vitro, (Araya J et al., manuscript in submission) and decreased PARK2 levels (not PINK1 levels) were associated with lung function of COPD patients, suggesting that decreased PARK2 plays pivotal roles in COPD pathogenesis.

### 3.4. Mitochondria Biogenesis in COPD

What regulates the quality of mitochondria is not only mitochondrial elimination by mitophagy but also mitochondria biogenesis. Mitochondrial biogenesis is mainly regulated by the AMP-activated protein kinase (AMPK)-PGC1α pathway [45]. This pathway is stimulated by oxidative stresses. 

Cigarette smoke increases AMPK expression in bronchial epithelial cells [46]. AMPK phosphorylates multiple downstream targets and, hence, plays multiple roles in cell metabolism and signal transduction. AMPK activation caused premature senescence in fibroblasts [47] and increased cigarette smoke-induced IL-6 and IL-8 production (which are well-known senescence-associated secretory phenotype (SASP) factors [46]) in lung epithelial cells, while AMPK inhibited the cellular senescence in bronchial epithelial cell lines [48]. These bidirectional effects of AMPK on cellular senescence might be due to the multiple targets of AMPK, differences of cell type, and differences in concentration of cigarette smoke. Although AMPK plays some roles on COPD pathogenesis, the roles of mitochondria biogenesis in COPD were not shown in these studies. 

DNA damage response (DDR), inducible by cigarette smoke, increases PGC1α and mitochondrial biogenesis [35]. PGC-1α expression was markedly increased in mild COPD, [49], and PGC1α was upregulated in bronchial epithelial cells derived from COPD [42], suggesting that mitochondrial biogenesis is increased in COPD. Upregulation of mitochondria biogenesis itself is an adaptive response to oxidative stresses. PGC1α-induced mitochondrial biogenesis can restore intracellular mitochondrial function exposed to transient stress [34]. However, under prolonged stresses, increased mitochondria biogenesis might cause deleterious effects to cells. Indeed, DDR-induced mitochondria biogenesis with PGC1α upregulation induces cell senescence [35], and we recently found that increased mitochondrial biogenesis by PGC1β accelerated CSE-induced cellular senescence in human bronchial epithelial cells (Saito N et al., unpublished).

These observations suggest that the increased mitochondrial mass induced by prolonged stresses might play some roles in COPD pathogenesis. We hypothesized that newly generated healthy mitochondria exposed to prolonged stresses become damaged soon after their birth without improving intracellular mitochondrial functions, and increased damaged mitochondria with ROS induces cellular senescence. 

### 3.5. Mitochondria Function in COPD

Mitochondrial functions were altered in both bronchial epithelial cells [42] and airway smooth muscle (ASM) cells [50] derived from COPD patients. While oxidative phosphorylation complex V is increased, reflecting a compensatory mechanism in response to increased energy demand [42] and/or elevated oxidative stress in COPD bronchial epithelial cells [15], mitochondrial dysfunction with excessive ROS contributes to their pro-inflammatory and proliferative properties in COPD ASM cells [50]. Hoffmann et al. demonstrated increased numbers of mitochondria with cristae depletion in COPD epithelial cells [42], indicating that mitochondria in COPD are damaged. Mitochondrial damage induced by prolonged cigarette smoke exposure overwhelms quality control systems, leading to the accumulation of dysfunctional mitochondria in COPD. Mitochondrial ROS from damaged mitochondria play critical roles in CSE-induced cellular senescence in bronchial epithelial cells.

Effects of CSE on mitochondrial dynamics, mitophagy, and biogenesis in an in vitro and in vivo model depend on experimental conditions. In addition, some responses are adaptive responses, which are protective against cell fate change, while others are causal for cell fate. Hence, the precise evaluation of mitochondrial quality systems in COPD is difficult and further studies are needed to elucidate it.

## 4. IPF

### 4.1. Mitochondria Quality Control in IPF

**Figure 3:**
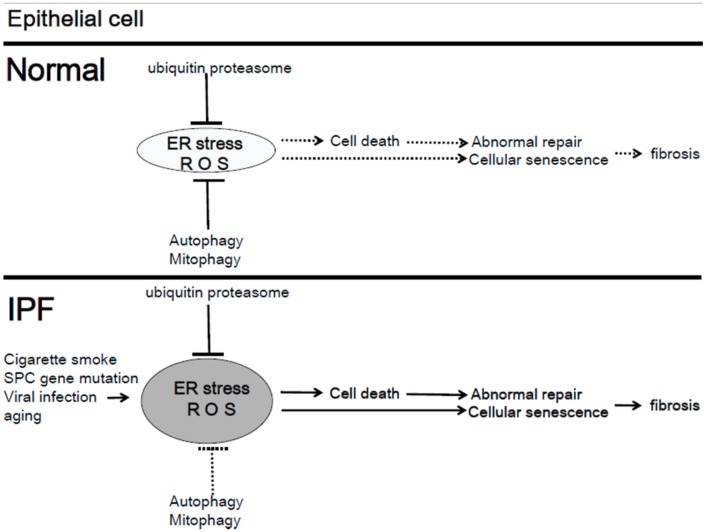
The role of endoplasmic reticulum (ER) stress, autophagy, and mitophagy in the pathogenesis of Idiopathic pulmonary fibrosis (IPF). In epithelial cells, excessive ER stress induces cell death and senescence. Dysregulated autophagy and mitophagy accelerate ER stress-induced cell death and senescence.

IPF is a chronic, progressive, and fibrotic lung disease [51]. Epithelial cells are exposed to both intracellular and extracellular stresses. Chronic stresses including abnormal surfactant accumulation, cigarette smoking, viral infection, and aging cause endoplasmic reticulum (ER) stress in epithelial cells in IPF [52,53]. ER stress induces cell death and senescence in the epithelial cell, leading to lung fibrosis in IPF (Figure 3). 

ER and mitochondria are closely linked [54]. The mitochondria-associated endoplasmic reticulum membrane (MAM) is a subdomain of the ER membrane that regulates ER-mitochondria communications. MAM plays important roles in calcium signaling, phospholipid biosynthesis, protein folding, and membrane tethering [55]. ROS induces calcium release from ER to the cytosol, and cytosolic calcium increases mitochondrial ROS production, forming a self-perpetuating loop. Increased ROS and perturbation of ER calcium homeostasis can disrupt the protein folding process, causing ER stress. Since damaged mitochondria increase both ROS production and ER stress, it is plausible that mitochondria quality control systems play some roles in IPF pathogenesis. Indeed, increasing evidence suggests that mitochondrial dysfunction plays important roles in the pathogenesis of IPF [51].

### 4.2. Mitochondrial Dynamics in IPF

As described above, ER stress is upregulated in epithelial cells in IPF. Aging is a major risk factor for IPF, and lung epithelial cells are senescent [56]. Hence, ER stress and senescence play critical roles in IPF pathogenesis. Alveolar epithelial cells derived from aged mice, in which ER stress in the lung induced by viral infection demonstrated an accelerated lung fibrosis with abnormally enlarged mitochondria [18]. Inactivated DRP1, as well as an enhanced expression of OPA1 and MFN1/2 in these cells indicating mitochondrial fusion, is predominant in this mouse model of IPF. Indeed, mitochondria in IPF alveolar epithelial cells were enlarged and dysmorphic with a significantly increased mitochondrial area, indicating that mitochondria fusion is predominant in IPF lung epithelial cells. In contrast with stress-induced mitochondrial hyperfusion, these mitochondria showed decreased functions.

There are no reports that demonstrate mitochondrial morphology in other types of cells in IPF. However, lung fibroblasts that mimic IPF fibroblasts through knockdown PARK2 showed elongated mitochondria with increased ATP production and proliferation [19]. This is consistent with fibroblast activation in IPF.

### 4.3. Mitophagy in IPF

#### 4.3.1. Mitophagy in Lung Epithelial Cells

In IPF, the dysregulation of both PINK1 and PARK2 has been reported. Bueno et al. demonstrated that PINK1 expression was downregulated in IPF alveolar epithelial cells [18]. In addition to decreased PINK1 expression, the colocalization of ATP synthase (mitochondrial protein) and LC3 was decreased in IPF epithelial cells, and dysfunctional large mitochondria with disorganized cristae were accumulated in these cells, indicating decreased mitophagy in IPF epithelial cells. In the mouse model exposed to increasing ER stress by a viral infection, PINK1 deficiency results in the accumulation of damaged mitochondria increased cell apoptosis and increased susceptibility to lung fibrosis. The upregulation of PINK1 by thyroid hormone reversed bleomycin-induced lung fibrosis, which might be due to restoring mitochondrial function through mitophagy [34]. This data suggested that PINK1 is protective against lung fibrosis, and decreased mitophagy due to PINK1 deficiency plays important roles in IPF pathogenesis. Elevated ER stress in epithelial cells is a possible cause of PINK1 deficiency. ER stress induces ATF3 expression, which binds to PINK1 and represses the transcription of PINK1, leading to the accumulation of dysfunctional mitochondria with increased mitochondrial ROS generation [57].

In contrast, TGF-β, a pro-fibrotic mediator increased in the BAL fluids of IPF patients, increased the PINK expression with increased colocalization of PINK1 with LC3 in a bronchial cell line [58]. Silencing PINK1 enhanced cell death in response to TGF-β suggests that PINK1 elevation might be a protective response to TGF-β. The effect of TGF-β on PINK1 induction might be insufficient in IPF.

Mitophagy alleviates ER stress-induced cell death or senescence by inhibiting excessive mitochondrial ROS production. Insufficient mitophagy in epithelial cells accelerates fibrosis development in IPF (Figure 3).

#### 4.3.2. Mitophagy in Lung Fibroblasts

In IPF fibroblasts, Kobayashi et al. reported that PARK2 expression is decreased compared to epithelial cells. Decreased PARK2 results in a decreased mitophagy and the subsequent increased mitochondrial ROS production. ROS activates PDGF receptors and downstream Akt and mTOR, leading to increased proliferation of fibroblasts and myofibroblast differentiation [19]. The activation of mTOR suppresses mitophagy, forming a self-perpetuating loop.

Not only PARK2 expression, but PINK1 expression is also decreased in IPF lung fibroblasts. PINK1 expression was non-detectable in the fibrotic foci [59], suggesting a decreased mitophagy in IPF fibroblasts. TGF-β represses mitophagy in lung fibroblasts and inhibits PINK1 expression during pulmonary fibrosis in both a mouse model and in vitro analysis.

In IPF, mitophagy might be suppressed in both epithelial cells and fibroblast and plays critical roles on IPF pathogenesis.

### 4.4. Mitochondria Biogenesis in IPF

Although increasing evidence suggests that mitophagic degradation of damaged mitochondria is decreased and dysfunctional mitochondria accumulate in IPF, the levels of mitochondrial biogenesis in IPF remains to be understood. Recently, Yu et al. demonstrated that PGC1α is decreased in patients with idiopathic pulmonary fibrosis, suggesting that mitochondrial biogenesis is decreased in IPF. They demonstrated that the upregulation of PGC1α by the thyroid hormone reversed bleomycin-induced lung fibrosis, which was attributed to restored mitochondrial function by increased mitochondrial biogenesis [34]. Mitochondrial biogenesis, in restoring mitochondrial function, might have anti-fibrotic effects in IPF. 

### 4.5. Other Mitochondrial Quality Control Systems in IPF

Mitochondrial NAD-dependent protein deacetylase, Sirtuin (SIRT) 3, maintains mitochondrial function by preventing the hyperacetylation of ETC complexes. Under stress conditions, Sirt3 promotes mitochondrial fusion by the deacetylation of OPA1 and maintains mitochondrial function [60]. SIRT3 deficiency promotes lung fibrosis by augmenting alveolar epithelial cell mitochondrial DNA damage and apoptosis [61]. Tissue from patients with IPF has shown increased acetylation of MnSOD^K68^, a known SIRT3 deacetylase target, suggesting that SIRT3 deficiency might play some role in IPF pathogenesis. 

## 5. Conclusions

Quality control systems of mitochondria, including mitochondria dynamics, mitophagy, and mitochondrial biogenesis, are critical for the maintenance of intracellular homeostasis. The dysregulation of these systems plays important roles in the development of COPD and IPF.

Elucidating the precise mechanisms of the involvement of mitochondrial quality control systems on these age-related lung diseases may lead to the development of new therapies for these diseases.

## References

[1] Shadel G.S., Horvath T.L. (2015). Mitochondrial ROS signaling in organismal homeostasis. Cell.

[2] Murphy E., Ardehali H., Balaban R.S., DiLisa F., Dorn G.W., Kitsis R.N., Otsu K., Ping P., Rizzuto R., Sack M.N. (2016). Mitochondrial Function, Biology, and Role in Disease: A Scientific Statement From the American Heart Association. Circ. Res..

[3] Ziegler D.V., Wiley C.D., Velarde M.C. (2015). Mitochondrial effectors of cellular senescence: Beyond the free radical theory of aging. Aging Cell.

[4] Zemirli N., Morel E., Molino D. (2018). Mitochondrial Dynamics in Basal and Stressful Conditions. Int. J. Mol. Sci..

[5] Ni H.M., Williams J.A., Ding W.X. (2015). Mitochondrial dynamics and mitochondrial quality control. Redox Biol..

[6] Kiriyama Y., Nochi H. (2017). Intra- and Intercellular Quality Control Mechanisms of Mitochondria. Cells.

[7] Shi R., Guberman M., Kirshenbaum L.A. (2018). Mitochondrial quality control: The role of mitophagy in aging. Trends Cardiovasc. Med..

[8] Larsen S.B., Hanss Z., Kruger R. (2018). The genetic architecture of mitochondrial dysfunction in Parkinson’s disease. Cell Tissue Res..

[9] Payne B.A., Chinnery P.F. (2015). Mitochondrial dysfunction in aging: Much progress but many unresolved questions. Biochim. Biophys. Acta.

[10] Bratic A., Larsson N.G. (2013). The role of mitochondria in aging. J. Clin. Investig..

[11] Sun N., Youle R.J., Finkel T. (2016). The Mitochondrial Basis of Aging. Mol. Cell.

[12] Ahlqvist K.J., Hamalainen R.H., Yatsuga S., Uutela M., Terzioglu M., Gotz A., Forsström S., Salven P., Angers-Loustau A., Kopra O.H. (2012). Somatic progenitor cell vulnerability to mitochondrial DNA mutagenesis underlies progeroid phenotypes in Polg mutator mice. Cell Metab..

[13] Ito S., Araya J., Kurita Y., Kobayashi K., Takasaka N., Yoshida M., Hara H., Minagawa S., Wakui H., Fujii S. (2015). PARK2-mediated mitophagy is involved in regulation of HBEC senescence in COPD pathogenesis. Autophagy.

[14] Mizumura K., Cloonan S.M., Nakahira K., Bhashyam A.R., Cervo M., Kitada T., Glass K., Owen C.A., Mahmood A., Washko G.R. (2014). Mitophagy-dependent necroptosis contributes to the pathogenesis of COPD. J. Clin. Investig..

[15] Hara H., Araya J., Ito S., Kobayashi K., Takasaka N., Yoshii Y., Wakui H., Kojima J., Shimizu K., Numata T. (2013). Mitochondrial fragmentation in cigarette smoke-induced bronchial epithelial cell senescence. Am. J. Physiol. Lung Cell. Mol. Physiol..

[16] Michaeloudes C., Bhavsar P.K., Mumby S., Chung K.F., Adcock I.M. (2017). Dealing with Stress: Defective Metabolic Adaptation in Chronic Obstructive Pulmonary Disease Pathogenesis. Ann. Am. Thorac. Soc..

[17] Aggarwal S., Mannam P., Zhang J. (2016). Differential regulation of autophagy and mitophagy in pulmonary diseases. Am. J. Physiol. Lung Cell. Mol. Physiol..

[18] Bueno M., Lai Y.C., Romero Y., Brands J., St Croix C.M., Kamga C., Corey C., Herazo-Maya J.D., Sembrat J., Lee J.S. (2015). PINK1 deficiency impairs mitochondrial homeostasis and promotes lung fibrosis. J. Clin. Investig..

[19] Kobayashi K., Araya J., Minagawa S., Hara H., Saito N., Kadota T., Sato N., Yoshida M., Tsubouchi K., Kurita Y. (2016). Involvement of PARK2-Mediated Mitophagy in Idiopathic Pulmonary Fibrosis Pathogenesis. J. Immunol..

[20] Song Z., Ghochani M., McCaffery J.M., Frey T.G., Chan D.C. (2009). Mitofusins and OPA1 mediate sequential steps in mitochondrial membrane fusion. Mol. Biol. Cell.

[21] Smirnova E., Griparic L., Shurland D.L., van der Bliek A.M. (2001). Dynamin-related protein Drp1 is required for mitochondrial division in mammalian cells. Mol. Biol. Cell.

[22] Tondera D., Grandemange S., Jourdain A., Karbowski M., Mattenberger Y., Herzig S., Da Cruz S., Clerc P., Raschke I., Merkwirth C. (2009). SLP-2 is required for stress-induced mitochondrial hyperfusion. EMBO J..

[23] Youle R.J., van der Bliek A.M. (2012). Mitochondrial fission, fusion, and stress. Science.

[24] Ehses S., Raschke I., Mancuso G., Bernacchia A., Geimer S., Tondera D., Martinou J.C., Westermann B., Rugarli E.I., Langer T. (2009). Regulation of OPA1 processing and mitochondrial fusion by m-AAA protease isoenzymes and OMA1. J. Cell Biol..

[25] Ahmad T., Sundar I.K., Lerner C.A., Gerloff J., Tormos A.M., Yao H., Rahman I. (2015). Impaired mitophagy leads to cigarette smoke stress-induced cellular senescence: Implications for chronic obstructive pulmonary disease. FASEB J..

[26] Lee S., Jeong S.Y., Lim W.C., Kim S., Park Y.Y., Sun X., Youle R.J., Cho H. (2007). Mitochondrial fission and fusion mediators, hFis1 and OPA1, modulate cellular senescence. J. Biol. Chem..

[27] Mizushima N., Komatsu M. (2011). Autophagy: Renovation of cells and tissues. Cell.

[28] Kuwano K., Araya J., Hara H., Minagawa S., Takasaka N., Ito S., Kobayashi K., Nakayama K. (2016). Cellular senescence and autophagy in the pathogenesis of chronic obstructive pulmonary disease (COPD) and idiopathic pulmonary fibrosis (IPF). Respir. Investig..

[29] Pickles S., Vigie P., Youle R.J. (2018). Mitophagy and Quality Control Mechanisms in Mitochondrial Maintenance. Curr. Biol..

[30] Zhang C., Yu X., Gao J., Zhang Q., Sun S., Zhu H., Yang X., Yan H. (2018). PINK1/Parkin-mediated mitophagy was activated against 1,4-Benzoquinone-induced apoptosis in HL-60 cells. Toxicol. In Vitro.

[31] Rambold A.S., Kostelecky B., Elia N., Lippincott-Schwartz J. (2011). Tubular network formation protects mitochondria from autophagosomal degradation during nutrient starvation. Proc. Natl. Acad. Sci. USA.

[32] Palikaras K., Lionaki E., Tavernarakis N. (2015). Balancing mitochondrial biogenesis and mitophagy to maintain energy metabolism homeostasis. Cell Death Differ..

[33] Kauppila T.E.S., Kauppila J.H.K., Larsson N.G. (2017). Mammalian Mitochondria and Aging: An Update. Cell Metab..

[34] Yu G., Tzouvelekis A., Wang R., Herazo-Maya J.D., Ibarra G.H., Srivastava A., De Castro J.P.W., DeIuliis G., Ahangari F., Woolard T. (2018). Thyroid hormone inhibits lung fibrosis in mice by improving epithelial mitochondrial function. Nat. Med..

[35] Correia-Melo C., Marques F.D., Anderson R., Hewitt G., Hewitt R., Cole J., Carroll B.M., Miwa S., Birch J., Merz A. (2016). Mitochondria are required for pro-ageing features of the senescent phenotype. EMBO J..

[36] Yin X.M., Ding W.X. (2013). The reciprocal roles of PARK2 and mitofusins in mitophagy and mitochondrial spheroid formation. Autophagy.

[37] Tsuji T., Aoshiba K., Nagai A. (2006). Alveolar cell senescence in patients with pulmonary emphysema. Am. J. Respir. Crit. Care Med..

[38] Demedts I.K., Demoor T., Bracke K.R., Joos G.F., Brusselle G.G. (2006). Role of apoptosis in the pathogenesis of COPD and pulmonary emphysema. Respir. Res..

[39] Yue L., Yao H. (2016). Mitochondrial dysfunction in inflammatory responses and cellular senescence: Pathogenesis and pharmacological targets for chronic lung diseases. Br. J. Pharmacol..

[40] Ballweg K., Mutze K., Konigshoff M., Eickelberg O., Meiners S. (2014). Cigarette smoke extract affects mitochondrial function in alveolar epithelial cells. Am. J. Physiol. Lung Cell. Mol. Physiol..

[41] Van der Toorn M., Rezayat D., Kauffman H.F., Bakker S.J., Gans R.O., Koeter G.H., Choi A.M., van Oosterhout A.J., Slebos D.J. (2009). Lipid-soluble components in cigarette smoke induce mitochondrial production of reactive oxygen species in lung epithelial cells. Am. J. Physiol. Lung Cell. Mol. Physiol..

[42] Hoffmann R.F., Zarrintan S., Brandenburg S.M., Kol A., de Bruin H.G., Jafari S., Dijk F., Kalicharan D., Kelders M., Gosker H.R. (2013). Prolonged cigarette smoke exposure alters mitochondrial structure and function in airway epithelial cells. Respir. Res..

[43] Kanaji N., Basma H., Nelson A., Farid M., Sato T., Nakanishi M., Wang X., Michalski J., Li Y., Gunji Y. (2014). Fibroblasts that resist cigarette smoke-induced senescence acquire profibrotic phenotypes. Am. J. Physiol. Lung Cell. Mol. Physiol..

[44] Soulitzis N., Neofytou E., Psarrou M., Anagnostis A., Tavernarakis N., Siafakas N., Tzortzaki E.G. (2012). Downregulation of lung mitochondrial prohibitin in COPD. Respir. Med..

[45] Zhang Z., Cheng X., Yue L., Cui W., Zhou W., Gao J., Yao H. (2018). Molecular pathogenesis in chronic obstructive pulmonary disease and therapeutic potential by targeting AMP-activated protein kinase. J. Cell. Physiol..

[46] Tang G.J., Wang H.Y., Wang J.Y., Lee C.C., Tseng H.W., Wu Y.L., Shyue S.K., Lee T.S., Kou Y.R. (2011). Novel role of AMP-activated protein kinase signaling in cigarette smoke induction of IL-8 in human lung epithelial cells and lung inflammation in mice. Free. Radic. Biol. Med..

[47] Wang W., Yang X., Lopez de Silanes I., Carling D., Gorospe M. (2003). Increased AMP:ATP ratio and AMP-activated protein kinase activity during cellular senescence linked to reduced HuR function. J. Biol. Chem..

[48] Cheng X.Y., Li Y.Y., Huang C., Li J., Yao H.W. (2017). AMP-activated protein kinase reduces inflammatory responses and cellular senescence in pulmonary emphysema. Oncotarget.

[49] Li J., Dai A., Hu R., Zhu L., Tan S. (2010). Positive correlation between PPARgamma/PGC-1alpha and gamma-GCS in lungs of rats and patients with chronic obstructive pulmonary disease. Acta Biochim. Biophys. Sin..

[50] Wiegman C.H., Michaeloudes C., Haji G., Narang P., Clarke C.J., Russell K.E., Bao W., Pavlidis S., Barnes P.J., Kanerva J. (2015). Oxidative stress-induced mitochondrial dysfunction drives inflammation and airway smooth muscle remodeling in patients with chronic obstructive pulmonary disease. J. Allergy Clin. Immunol..

[51] Zank D.C., Bueno M., Mora A.L., Rojas M. (2018). Idiopathic Pulmonary Fibrosis: Aging, Mitochondrial Dysfunction, and Cellular Bioenergetics. Front. Med..

[52] Burman A., Tanjore H., Blackwell T.S. (2018). Endoplasmic reticulum stress in pulmonary fibrosis. Matrix Biol..

[53] Korfei M., Ruppert C., Mahavadi P., Henneke I., Markart P., Koch M., Lang G., Fink L., Bohle R.M., Seeger W. (2008). Epithelial endoplasmic reticulum stress and apoptosis in sporadic idiopathic pulmonary fibrosis. Am. J. Respir. Crit. Care Med..

[54] Zhang K. (2010). Integration of ER stress, oxidative stress and the inflammatory response in health and disease. Int. J. Clin. Exp. Med..

[55] Fujimoto M., Hayashi T. (2011). New insights into the role of mitochondria-associated endoplasmic reticulum membrane. Int. Rev. Cell Mol. Biol..

[56] Minagawa S., Araya J., Numata T., Nojiri S., Hara H., Yumino Y., Kawaishi M., Odaka M., Morikawa T., Nishimura S.L. (2011). Accelerated epithelial cell senescence in IPF and the inhibitory role of SIRT6 in TGF-beta-induced senescence of human bronchial epithelial cells. Am. J. Physiol. Lung Cell. Mol. Physiol..

[57] Bueno M., Brands J., Voltz L., Fiedler K., Mays B., St Croix C., Sembrat J., Mallampalli R.K., Rojas M., Mora A.L. (2018). ATF3 represses PINK1 gene transcription in lung epithelial cells to control mitochondrial homeostasis. Aging Cell.

[58] Patel A.S., Song J.W., Chu S.G., Mizumura K., Osorio J.C., Shi Y., El-Chemaly S., Lee C.G., Rosas I.O., Elias J.A. (2015). Epithelial cell mitochondrial dysfunction and PINK1 are induced by transforming growth factor-beta1 in pulmonary fibrosis. PLoS ONE.

[59] Sosulski M.L., Gongora R., Danchuk S., Dong C., Luo F., Sanchez C.G. (2015). Deregulation of selective autophagy during aging and pulmonary fibrosis: The role of TGFbeta1. Aging Cell.

[60] Samant S.A., Zhang H.J., Hong Z., Pillai V.B., Sundaresan N.R., Wolfgeher D., Archer S.L., Chan D.C., Gupta M.P. (2014). SIRT3 deacetylates and activates OPA1 to regulate mitochondrial dynamics during stress. Mol. Cell. Biol..

[61] Jablonski R.P., Kim S.J., Cheresh P., Williams D.B., Morales-Nebreda L., Cheng Y., Yeldandi A., Bhorade S., Pardo A., Selman M. (2017). SIRT3 deficiency promotes lung fibrosis by augmenting alveolar epithelial cell mitochondrial DNA damage and apoptosis. FASEB J..

